# Core health indicators in countries with high proportion of expatriates: Case study of Qatar

**DOI:** 10.3389/fpubh.2023.1035686

**Published:** 2023-02-07

**Authors:** Maziar Moradi-Lakeh, Amine Toumi, Shams Eldin Khalifa, Henry Victor Doctor, Salah Alyafei, Sara Khamis Al Hamad, Mohammed Al-Thani, Arash Rashidian

**Affiliations:** ^1^Preventive Medicine and Public Health Research Center, Psychosocial Health Research Institute, Iran University of Medical Sciences, Tehran, Iran; ^2^Optimax Access LLC, Mission Viejo, CA, United States; ^3^Public Health Department, Ministry of Public Health, Doha, Qatar; ^4^Department of Science, Information and Dissemination, Regional Office for the Eastern Mediterranean, World Health Organization, Cairo, Egypt; ^5^Mortuary, Hamad Medical Center, Doha, Qatar

**Keywords:** expatriate, health indicator, migrant workers, Qatar, death registration, age standardization

## Abstract

**Background:**

Population size and structure have a huge impact on health indicators. In countries with a high proportion of expatriates, there are some limitations in estimating, aggregating and reporting of the health indicators, and corrections may be required in the established estimation methodologies. We review the case of Qatar to see how its specific population characteristics affect its health indicators.

**Methods:**

We used routinely collected data and reviewed and calculated a selected list of health indicators for Qatari and non-Qatari populations residing in Qatar. Mortality and cancer incidence rates, stratified by nationality, were used for this purpose. Also, a direct method was used to estimate completeness of the death registry, compared to the mortuary data.

**Results:**

Age and sex distribution of Qatari and non-Qatari populations are completely different. Compared to the mortuary data, completeness of death registration for the total population was estimated at 98.9 and 94.3%, with and without considering overseas deaths, respectively. Both estimates were considerably higher than estimates from the indirect methods. Mortality patterns were different even after standardization of age and stratification of sex groups; male age-standardized mortality rates were 502.7 and 242.3 per 100,000 individuals, respectively for Qataris and non-Qataris. The rates were closer in female populations (315.6 and 291.5, respectively). The leading types of cancer incidents were different in Qataris and non-Qataris.

**Conclusions:**

Expatriates are a dynamic population with high-turnover, different from Qatari population in their age-sex structure and health status. They are more likely to be young or middle-aged and are less affected by age related diseases and cancers. Also, they might be at higher risks for specific diseases or injuries. Aggregating indicators of Qatari and non-Qatari populations might be mis-leading for policy making purposes, and common estimation correction approaches cannot alleviate the limitations. High-proportion of expatriate population also imposes significant errors to some of the key demographic estimates (such as completeness of death registry). We recommend a standardized approach to consider nationality in addition to age and sex distributions for analysis of health data in countries with a high proportion of expatriates.

## Background

Population size and structure have a huge impact on development and health indicators; they are also necessary inputs for calculating or estimating many of the health-related indicators (1). Determinants of population, such as fertility, mortality and migration, are not only some of the important health indicators, but also affect measures of other health indicators through changing size and structure of the population (2–4). On the one hand, aging and reduced fertility affect working capacity of countries. On the other hand, some of the countries experience rapid development and need extra working resources to respond to their rapid growth. Various strategies are used by different countries to balance their populations with their current and future needs. These strategies include pro-natalist policies, liberal immigration policies, replacement migration and changing the retirement age (5, 6). Some of the countries which have a small national working population to match their increasing human resource needs, permit expatriates to live and work in their countries, usually for a limited period of time. This strategy enables recruitment of human resources, knowledge transfer and is a source of remittance for some countries. Qatar is one of the countries with the highest proportion of expatriates (i.e., non-Qatari individuals who live in Qatar) (7).

When the ratio of migrants to the total residents of a country is high, it can influence the health status of host countries in different ways such as a mass displacement of the population. For example, a natural disaster or war in a neighboring country can threaten the resilience of health system in the host country and affect its capacity to respond to population needs (8). This issue is not the case in countries like Qatar that have a planned strategy to accept labor expatriates. However, demographic and health characteristics of expatriates could be different from national residents. In Qatar, many of the expatriates are “healthy workers” with majority of young men from different regions of the world, especially south Asia, the Middle East and North Africa (9, 10). This creates a challenge with monitoring population health in that aggregating expatriates and national residents ends up with unexpected measures in health indicators. The expatriates' population is very dynamic; many of them stay in the host county for short periods of time, even less than a year and might be replaced by other individuals throughout the same year. Even if the host countries accurately count all health events within the expatriate population (such as incident cases of a disease), the size and characteristics of the at-risk population is continuously changing (11). Expatriates might have specific health needs, be vulnerable to specific health conditions, and less prone to some health conditions. However, we did not find any previous study specifically focused on the impact of characteristics of expatriates' health on the health indicators of the host countries. In Qatar, “Data Driven Intelligence” is an important enabling factor for the Public Health Strategies (PHS) (12). Monitoring of health indicators is a key part of the data driven intelligence. In this report, we reviewed the case of Qatar to see how its specific population characteristics, with a high proportion of expatriates, affect its health indicators.

## Methods

This study was a secondary analysis of the prospectively collected routine health data of Qatar. In a collaboration between the Ministry of Public Health in Qatar (MoPH) and the Eastern Mediterranean Regional Office (EMRO) of the World Health Organization (WHO), we prepared a list of all indicators from the following sources: Health-related indicators of Sustainable Development Goals (SDGs) (13), WHO Global Reference list for core health indicators (2), WHO Regional core health indicators (14), Qatar National Health Report (15) and the WHO 13th General Programme of Work (GPW 13) Impact Framework (3). A manual, entitled as “Core Health Indicators—Manual for data collection and processing, measuring or estimating indicators and regular publishing”, was developed for this purpose. The manual contained the following sections: Introduction; Timetable; Metadata (definitions; categorization; Methods and frequency of measurement or estimation; Internationally preferred sources of data; and Sources of data in Qatar), Sources of data by the custodian organizations; Relevant Health surveys; Formulae for calculating indicators; Stratifiers; Supplementary material and References. The Health Intelligence and Information Section (HIIS) in the MoPH contributed to the development of this manual, and it was also rapidly reviewed during a workshop by potential users in other organizations. The manual was being reviewed by a group of the final users. From the list, we selected the following (group of) indicators to assess how population characteristics of Qatar, specifically its high proportion of expatriates affect the measures:

- Mortality rates: Crude death rate, age-specific mortality rates and age-standardized mortality rates are some of the key health indicators, and are directly or indirectly related to several other health indicators, such as life expectancy, maternal mortality ratio, infant mortality rate, under 5 mortality rate and premature mortality of non-communicable diseases. Whenever estimation of mortality indicators is based on death registry, its completeness needs to be considered in the calculation process. In this study, completeness of death registration in Qatar was estimated by comparing registered deaths at MoPH with mortuary data for 2018. Based on current rules and regulations, all cases of death in Qatar, both for Qatari nationals and non-Qataris, pass through the mortuary before burial in Qatar or repatriation to their countries of origin. Supplementary Figure S1 shows the process which is followed when a Qatari or non-Qatari dies in Qatar, or an individual with Qatari nationality passes away outside the country. Individual-level data (of deceased cases) or aggregated data based on customized age categories and nationality are not publicly available in Qatar. For the purpose of this study, individual level data were received and analyzed at the MoPH. Since the counts of deaths in some of the age-sex-nationality population groups were low, 10-year age groups were used to provide more reliable estimates of mortality rates. The only exception was for under 5-year age group which was considered separately because of its higher risk of death. Our estimates of completeness were then compared to the previously reported estimates by the Death Distribution Methods (16, 17). Death distribution methods, such as the Generalized Growth Balance and Bennett-Horiuchi methods indirectly estimate completeness of death registration. These methods have also been used to estimate completeness of death registration in Qatar; and they have strong assumptions on stability of population and zero (or very low) migration.
- Cancer incidence: Since its establishment in 2014, Qatar National Cancer Registry (QNCR) has systematically and prospectively collected cancer data from all healthcare providers and sectors (18). The cancer incidence and mortality reported by QNCR were compared to the estimates of GLOBOCAN project (19, 20) and the Global Burden of Disease (GBD 2019) study (21). Both recent set of estimates are based on the available data and modeling approaches.

Data on population of Qatar in 2018 (by age-group, sex and nationality) were received from the Planning and Statistics Authority.

Age-standardization, and subgroup analyses based on nationality and sex were used for providing comparable measures. There was no need for any specific statistical test.

All methods were carried out in accordance with relevant guidelines and regulations of “Qatar supreme council of health guidelines, regulations and policies for research involving human subjects” (22). The study was exempted from ethical approval because no human subjects were involved in the study; and all the data were anonymous.

## Results

**Figure 1:**
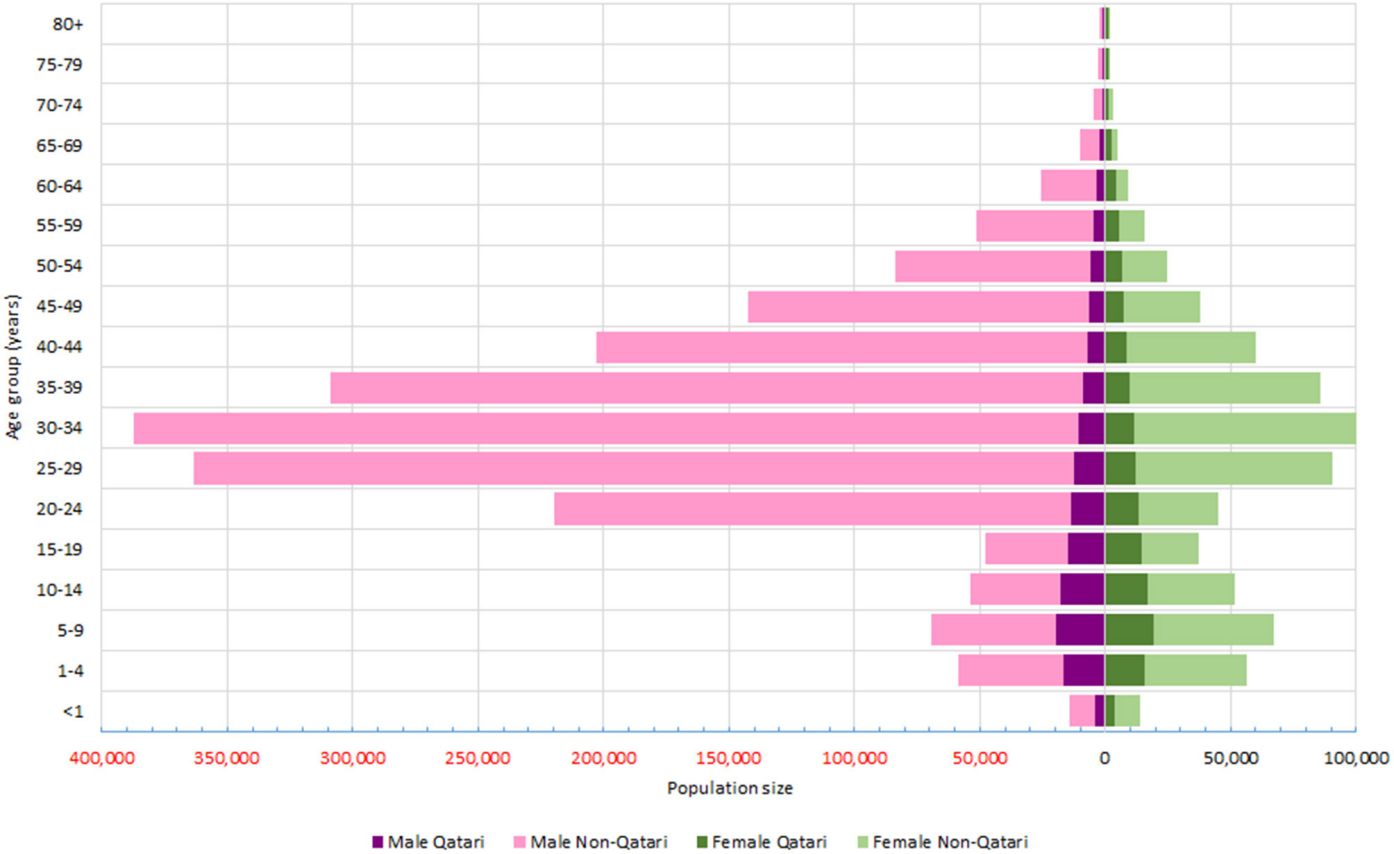
Population pyramid of Qatar by sex, age groups and nationality (2018).

Qatar population was 2,760,170 in 2018, with a male to female ratio of 2.88 and a non-Qatari to Qatari ratio of 7.95. Figure 1 displays the population structure of Qatar by age-group, sex and nationality. In 2018, 2,385 deaths were registered by the MoPH death registry. Of the deaths, 2,274 were related to deaths inside the country and 111 deaths were related to Qataris who died outside Qatar. During the same year, 2,412 deaths were documented in the mortuary system. Comparing to the mortuary data as the reference for number of deaths, MoPH death registry has a completeness of 98.2%.

Table 1 shows the number of deaths based on different datasets by sex and nationality, as well as completeness for each subgroup. Based on the MoPH inclusive data (which contains Qataris' deaths inside and outside the country), male to female ratio and non-Qatari to Qatari ratio for deaths were 2.61 and 2.15, respectively. All-age mortality rates were considerably higher in Qataris compared to non-Qatari population (Table 2). The ratio of mortality rates among non-Qataris to Qataris were 0.22, 0.34, and 0.27 for male, female and both sexes, respectively; they were 0.48, 0.92, and 0.64, respectively after age-standardization. In other words, Qatari males had much higher mortality than non-Qataris even after age-standardization, while female Qataris and non-Qataris were closer after age-standardization.

**Table 1:** Completeness of death registry at Ministry of Public Health (MoPH) compared to mortuary data, 2018.

**Measure**		**Count**			**Completeness**	
**Source of data**		**MoPH registry**, inside^*^	**MoPH registry**, inclusive^**^	**Mortuary**	**MoPH registry**, inside^*^	**MoPH registry**, inclusive^**^
Qatari	Male	388	455	446	87.0%	102.0%
	Female	257	301	294	87.4%	102.4%
	Total	645	756	742	86.9%	101.9%
Non-Qatari	Male	1,270	1,270	1,290	98.4%	98.4%
	Female	359	359	371	96.8%	96.8%
	Total	1,629	1,629	1,670	97.5%	97.5%
All	Male	1,658	1,725	1,736	95.5%	99.4%
	Female	616	660	665	92.6%	99.2%
	Total	2,274	2,385	2,412	94.3%	98.9%

* Includes deaths happened inside Qatar,

** Includes deaths of Qataris inside or outside the country.

**Table 2:** Mortality rates per 100,000 population in Qatar by age-group, sex and nationality, 2018.

**Age groups**	**Qatari**			**Non-Qatari**			**Total**		
	**Male**	**Female**	**Total**	**Male**	**Female**	**Total**	**Male**	**Female**	**Total**
0–4	159.3	151.7	155.6	147.0	144.2	145.6	150.5	146.3	148.5
5–14	16.1	22.0	19.0	7.0	8.5	7.7	9.8	12.6	11.2
15–24	173.1	21.3	98.3	31.5	22.0	29.7	46.8	21.8	40.9
25–34	99.8	16.8	57.5	32.7	15.8	29.5	34.8	15.9	30.9
35–44	137.6	98.1	116.5	50.6	14.9	43.3	53.3	25.4	47.1
45–54	375.7	177.6	269.7	103.3	74.3	97.9	118.1	97.5	113.6
55–64	891.3	471.9	660.5	270.0	257.5	267.7	334.4	340.2	335.9
65–74	1,993.6	1,223.8	1,577.9	994.4	1,710.0	1,183.2	1,226.7	1,467.8	1,312.0
75+	6,397.0	4,942.0	5,636.6	3,724.0	4,840.8	4,130.9	4,892.1	4,901.2	4,896.1
All ages	299.9	192.1	245.1	66.9	64.7	66.4	84.1	92.7	86.3
Age-standardized	502.7	315.6	403.8	242.3	291.5	259.0	304.0	295.3	301.4

The latest reported cancer incidence was 59.8 per 100,000 population at risk based on QNCR for 2016, and the age-standardized incidence rate (ASIR) was 135 per 100,000 population. The total number of cancers (malignant and *in-situ*) registered at QNCR in 2016 were 1,566, with a male to female ratio of 1.40 and non-Qatari to Qatari ratio of 3.69. Table 3 compares the GLOBOCAN and GBD 2017 estimates for Qatar with the QNCR measures. In addition to the rates of cancer, there were differences in the list and proportional share of the top 5 cancers with highest incidence rates among Qatari and non-Qatari sub-populations (Table 4).

**Table 3:** Incidence rates of cancers in Qatar based on Qatar National Cancer Registry (QNCR) and estimations of GBD 2019 and GLOBOCAN 2018.

**Age-group**	**QNCR** ^a^			**GBD2019^b^** **total** ***N***	**GLOBOCAN**^c^ **Total** **Age-group** ***N*** **(Rate)**	
	**Total** ***N*** **(Rate)**	**Qatari** ***N*** **(Rate)**	**Non-Qatari** ***N*** **(Rate)**			
0–4	20 (15.2)	9 (22.7)	12 (12.2)	22	0–14: (7.8)	
5–9	16 (12.9)	5 (13.1)	11 (12.9)	18		
10–14	5 (5.2)	2 (6.2)	3 (4.7)	21		
15–19	12 (13.5)	4 (14.2)	8 (13.1)	19	15–29: (10.3)	
20–24	24 (8.8)	8 (29.9)	16 (6.5)	46		
25–29	65 (14.9)	14 (59.0)	55 (12.5)	82		
30–34	130 (30.3)	15 (72.7)	118 (28.2)	126	30–44: (25.7)	
35–39	154 (47.4)	11 (63.1)	147 (46.5)	161		
40–44	159 (65.1)	17 (114.5)	144 (61.9)	161		
45–49	190 (108.9)	29 (216.7)	159 (99.9)	205	45-59: (108.9)	
50–54	183 (166.5)	36 (315.0)	145 (149.0)	189		
55–59	188 (259.3)	41 (438.8)	142 (232.0)	214		
60–64	160 (479.8)	38 (581.2)	119 (454.4)	177	60-74: (475.8)	
65-69	132 (890.0)	47 (1,273.7)	83 (760.3)	139		
70–74	67 (993.9)	24 (838.9)	43 (1,108.2)	93		70+: (990.2)
75-79	30 (753.8)	12 (567.6)	18 (964.6)	60		
80+	31 (934.9)	22 (1,324.5)	9 (543.8)	47		
Total	1,566 (59.8)	334 (114.1)	1,232 (53.0)	1,779	1,260 (46.8)	
Age-standardized	N/A (135.0)	N/A (186)	N/A (126)	N/A	N/A (97.3)	

a For the reference year of 2016,

b Recalculated based on GBD 2019 estimates for the reference year of 2016; B.1.30 (Other neoplasms) were excluded,

c For the reference year of 2018.

**Table 4:** Most common types of cancers by sex and nationality in Qatar, Qatar National Cancer Registry, 2016.

**Female non-Qataris**	**Female Qataris**	**Male non-Qataris**	**Male Qataris**
1 Breast, 191 (41.70)^*^ 2 Thyroid gland, 40 (8.73) 3 Uterus, 31 (6.77) 4 Non-Melanoma skin cancer, 30 (6.55) 5 Colorectal, 27 (5.90)	1 Breast, 68 (35.05) 2 Colorectal, 23 (11.86) 3 Thyroid gland, 17 (8.76) 4 Uterus, 16 (8.25) 5 Cervix, 12 (6.19)	1 Colorectal, 86 (11.11) 2 Non-Melanoma skin cancer, 69 (8.91) 3 Leukemia, 67 (8.66) 4 Prostate, 65 (8.40) 5 Non-Hodgkin Lymphoma, 37 (4.78)	1 Colorectal, 17 (12.14) 2 Prostate, 17 (12.14) 3 Leukemia, 12 (8.57) 4 Non-Hodgkin Lymphoma, 10 (7.14) 5 Bladder, 9 (6.43)

* Count (Percent).

GBD 2019 and GLOBOCAN 2018 do not provide stratified data by nationality of residents within a country.

## Discussion

In this study, estimates of mortality and cancer data were reviewed by sex, age group and nationality of the population of Qatar. Although the population structure of Qataris and non-Qataris are different by age and sex distribution, this is not the only reason for their different health status. Even after separate analyses by sex, standardized by age, the health status of Qataris and non-Qataris is not similar. The difference between male Qataris and non-Qataris is more evident, and non-Qatari males have lower mortality rates than Qatari males. However, there might be specific injuries or diseases which are more common in non-Qataris. This finding is consistent with another study that revealed higher age-standardized mortality rate in Qatari adults aged 20 years or older, compared to non-Qataris during the 1989–2015 period (23). This sounds reasonable because of the healthy workers' effect: individuals with chronic health conditions or disabilities are less likely to seek employment in another country, even for non-manual and office-based works. Men are the majority of expatriates and some of them bring their families to Qatar; their family members do not necessarily follow the same healthy workers' pattern. This might be the reason for the closer age-standardized indicators in Qatari and non-Qatari females compared to males. Age-specific mortality rates in Qatari and non-Qatari children were closer in this study compared to adults. A previous study showed marginally less infant mortality rates in Qataris compared to non-Qataris in the period of 2004–2014 (relative risk of mortality: 0.81, 95% confidence interval: 0.66–1.00) (24), however, our findings did not support it.

This study also revealed the difference in the incidence of cancers based on QNCR reports and the estimations of international reports, such as GLOBOCAN. According to Annex A of the GLOBOCAN report, to estimate 2018 incidence rates for Qatar, incidence rates of 2008–2012 for Qataris, were applied to the 2012 Qatari population and then, rates of non-Kuwaiti residents of Kuwait (2008–2012) were applied to the 2012 non-Qatari population. The final 2012 estimate for the total population was applied to the estimated population of Qatar in 2018 (19, 20). Such methods are frequently used for global health estimates, especially for countries with limited data. However, they impose lots of uncertainty that should be considered. They also might ignore several specific demographic and epidemiologic characteristics of each country. Regular publishing of the key health indicators such as cancer incidence by the national health authorities, stratified by key factors (such as age, sex and nationality), will reduce non-robust estimates by international organizations.

Compared with mortuary data, completeness of the MoPH death registry was very high, especially when deaths of Qataris occurring outside the country were included. This is different from reports of some international agencies that estimated completeness of death registry in Qatar around 55%(17), or the GBD study with a completeness of 87 and 72% for under 5 and 15–59 year olds, respectively, in 2017 (25, 26). These low estimates of completeness originate from the death distribution methods which use the demographic balancing equation. These methods have strong assumptions on migration and misreporting of age (25), and obviously are not suitable for countries like Qatar with its unique age-sex structure, dynamic population and high rates of immigration.

National and non-national residents of Qatar have different demographic and health characteristics. Reporting indicators for the whole population reflects the status quo, but might be misleading for policy making. This status needs specific analytic approach to keep health indicators useful for monitoring health outcomes and development of evidence-informed policies. Since the age and sex distribution of Qataris and non-Qataris is significantly different, age-standardization makes them comparable. Age-standardization of the total population of residents leads to generation of internationally comparable indicators (as required for many of the internationally introduced sets of indicators). However, it is not enough helpful for policy making and decisions. Aggregating Qatari and non-Qatari populations who have different health profiles and needs might be misleading. The high proportion of non-Qataris in the total population and their relatively better health status may mask the health needs Qataris. This is more obvious for males because most of the healthy workers' effect is related to this group. By looking at the trends for aggregated indicators, real changes might be simply underestimated, because the smaller but more stable Qatari population will be influenced by the large (but more dynamic) non-Qatari population. For instance, road traffic injuries (RTIs) have recently been among the top causes of deaths and disabilities in Qatar. Incidence of RTI is high in males (like in most other countries), and the incidences are higher in Qatari males than non-Qatari males of the same age (27). RTI deaths and incidence rates show decreasing trends recently and may be influenced by multiple interventions implemented by the State of Qatar such as speed cameras (28–30). However, without looking at stratified analysis by nationality, it would not be possible to assess the impact of interventions on the most vulnerable population for RTIs (young male Qataris).

On the other hand, like other important socio-economic stratifiers, disaggregated analysesby nationality are important for monitoring health equity. Expatriates may have specific health needs that are not be reflected within the internationally recommended health indicators. Such needs could be recognized by local experts with access to data and be addressed through relevant policies.

There are specific policy implications in countries with a high proportion of expatriates, both for public health authorities and development partners such as WHO. Countries need to establish specific processes for collection, analysis and reporting of data, and interpret them cautiously; otherwise, the indicators may mislead them for selecting policy options or assessing the impact of policies. It is also reasonable for the host countries with a high proportion of expatriates to consider additional health indicators to address specific health needs of expatriates. Development partners should consider this specific demographic pattern and its impact on estimating and interpreting national health indicators. Usual methods such as age-standardization might not be enough to make them useful for monitoring and decision making. The issue needs to be further discussed by experts of health metrics to achieve a feasible guideline for analysis of data and reporting of core health indicators in countries with high proportion of expatriates. This could be initiated as a joint activity by development partners and such countries.

## Strengths and limitations

The original health data of Qatar by age, sex, and nationality was used to discuss the validity of internationally estimated health indicators. These estimates usually do not consider the high proportion of expatriates in the population of Qatar or their different health profile compared to Qatari individuals.

We used the mortuary data as the reference, considering the data flow for deceased individuals in Qatar. Other methods such as capture-recapture could be used by other researchers to provide further information. There are potential factors such as accessibility, race, gender, and education that might influence health outcomes of different population subgroups, as individual or intersectional factors. We did not assess the root causes of differences in health outcomes between Qataris and non-Qataris. This could be a topic for further research.

## Conclusion

Measurement, estimation, reporting and interpreting of health indicators for countries with a high proportion of expatriates such as Qatar need specific considerations. Regular indirect statistical methods for estimating completeness of death registries lead to inaccurate estimates. Such countries need to stratify the indicators by nationality to avoid being misled by aggregated measures, and to monitor health equity. International organizations should consider this specific population structure which affect estimations beyond the age and sex structure of the population.

## Data availability statement

The original contributions presented in the study are included in the article/Supplementary material, further inquiries can be directed to the corresponding author.

## Author contributions

MM-L, HD, AR, SA, and MA-T conceptualized the idea. AT, SK, SA, and MM-L collected data. AT and MM-L analyzed the data. MM-L drafted the manuscript and all other authors critically appraised the draft, and contributed to finalizing the manuscript. All authors approved the final version.

## Supplementary material

Supplementary Figure S1 Flow of data and corpse bodies for cases of death inside Qatar (Qatari or non-Qatari), and Qatari individuals outside the country.
