# Supplementary figures and images for: Core health indicators in countries with high proportion of expatriates: Case study of Qatar
Source: Front Public Health. 2023 Feb 7;11:1035686. doi: 10.3389/fpubh.2023.1035686 (PMC9941695; doi:10.3389/fpubh.2023.1035686)

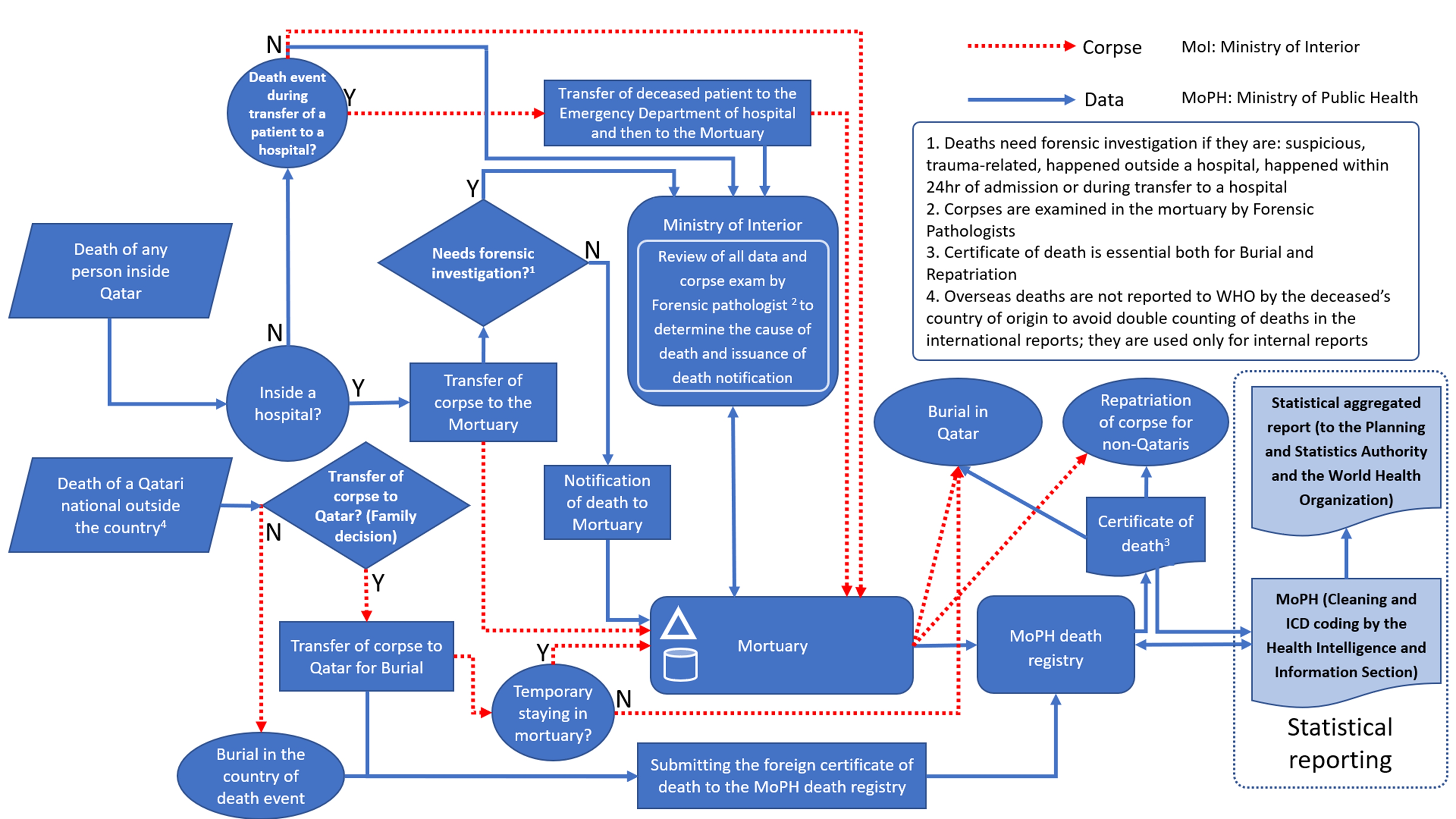

Supplement: Supplementary Figure S1 — Flow of data and corpse bodies for cases of death inside Qatar (Qatari or non-Qatari), and Qatari individuals outside the country. [file Image_1.tif]
